# Acquired punctate palmoplantar keratoderma following Programmed cell death protein 1 inhibitor therapy

**DOI:** 10.1016/j.jdcr.2025.10.030

**Published:** 2025-10-25

**Authors:** Nandini Sarkar, Janina Markidan, Mark Gibbs, Klaus Helm

**Affiliations:** a Department of Medical Education, Pennsylvania State College of Medicine, Hershey, Pennsylvania; b Department of Pathology, Lebanon VA Medical Center, Lebanon, Pennsylvania; c Department of Dermatology, Lebanon VA Medical Center, Lebanon, Pennsylvania; d Professor of Dermatology and Pathology, Penn State Health Milton S. Hershey Medical Center, Penn State University, Hershey, Pennsylvania

**Keywords:** dermatologic side effects, immune-related adverse effects, immunotherapy, PD-1 inhibitor, pembrolizumab, programmed death-1 receptor (PD-1), punctate palmoplantar keratoderma

## Introduction

Palmoplantar keratoderma (PPK) refers to a group of hereditary and acquired disorders characterized by abnormal thickening and hyperkeratosis of the palms and soles.^1^^,^^2^ Different types are classified based on the clinical pattern of involvement as diffuse, focal (pressure-associated), or punctate.^1^^,^^2^

Punctate PPK is a rare subtype with an estimated prevalence of 1.17 per 100,000 individuals.^2^ Clinically, punctate PPK presents as multiple small, keratotic papules with central depressions, often distributed along palmar creases.^1^, ^2^, ^3^, ^4^ Unlike focal PPK, these lesions are usually not associated with pressure points.^1^

Acquired punctate PPK may arise from various etiologies, including inflammatory conditions, infections, medications, and malignancy.^1^^,^^5^ It has been described as a paraneoplastic phenomenon associated with a range of malignancies, including esophageal, lung, breast, bladder, gastric, colon, and skin cancers.^1^ Drug-induced cases have been reported with agents such as glucan, tegafur, venlafaxine, targeted kinase inhibitors including MEK inhibitors and BRAF inhibitors, and quinacrine.^1^^,^^6^ Notably, quinacrine in combination with chemotherapeutic agents such as bleomycin and hydroxyurea has been linked to the development of hyperkeratosis.^1^ However, PPK remains a unique adverse event, particularly in the realm of immunotherapy medications.

Here, we describe a rare case of acquired punctate PPK in a patient treated with pembrolizumab, a programmed cell death protein 1 (PD-1) inhibitor, for metastatic recurrent colon cancer. Although a previous case study has described pembrolizumab-induced PPK,^7^ the punctate form of PPK specifically has yet to be reported in association with PD-1 inhibitor therapy.

## Case report

**Fig 1:**
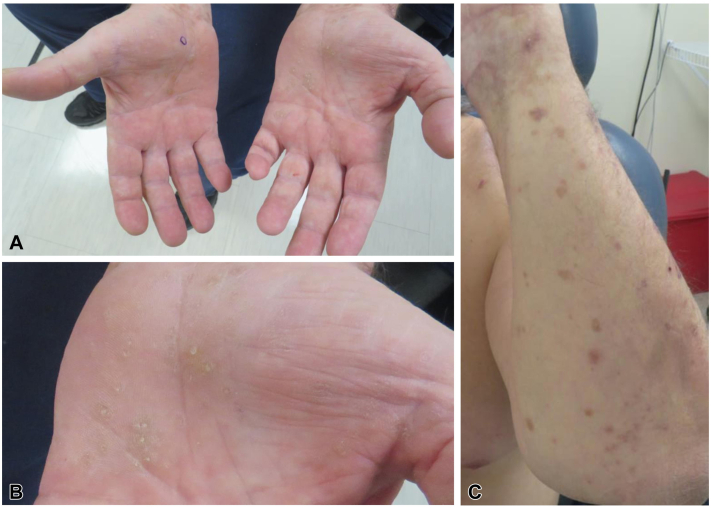
**A,** Physical examination of patient showing a bilateral cutaneous eruption on palmar surfaces. **B,** Close-up examination of lesions on the left hand, demonstrating small yellow papules with central invagination. **C,** Multiple scattered, violaceous plaques along the left forearm, consistent with clinical diagnosis of lichenoid reaction. Histopathological examination of the palm biopsy showed focal epidermal invagination with overlying compact orthohyperkeratosis, consistent with punctate PPK (Fig 2).^1^, ^2^, ^3^, ^4^, ^5^^,^^8^ Additionally, there was lichenoid interface inflammation with dyskeratotic keratinocytes and rare dermal eosinophils (Fig 3). The forearm biopsy showed findings consistent with a typical lichenoid drug eruption.

A 65-year-old male presented to the dermatology clinic with new onset of multiple keratotic yellow papules with central depressions on the palms (Fig 1, *A* and *B*), accompanied by violaceous, scaly plaques on the forearms (Fig 1, *C*), both persisting for several weeks. Scattered hyperpigmented macules were also noted on the forearms and dorsal feet. The plantar surfaces of the feet were spared.

The patient had a known history of Lynch syndrome due to a germline MSH2 mutation, along with pathogenic variants in PALB2 and BRCA2. He had been recently treated for metastatic recurrence of colorectal carcinoma, originally diagnosed and treated 25 years earlier. When the patient had his recurrence, he received 7 of his planned 24 cycles of bevacizumab and FOLFIRI (fluorouracil, leucovorin, and irinotecan) via IV infusion every 2 weeks. This was discontinued because he developed recurrent toxic encephalopathy.

Following termination of this regimen, he received 400 mg of pembrolizumab via IV infusion every 42 days. The medication was discontinued after 5 cycles due to pembrolizumab-induced hypothyroidism. Evaluation in the dermatology clinic occurred 6 months following initiation of pembrolizumab. At this time, shave biopsies were performed on lesions of the forearm and palmar hand.

**Fig 2:**
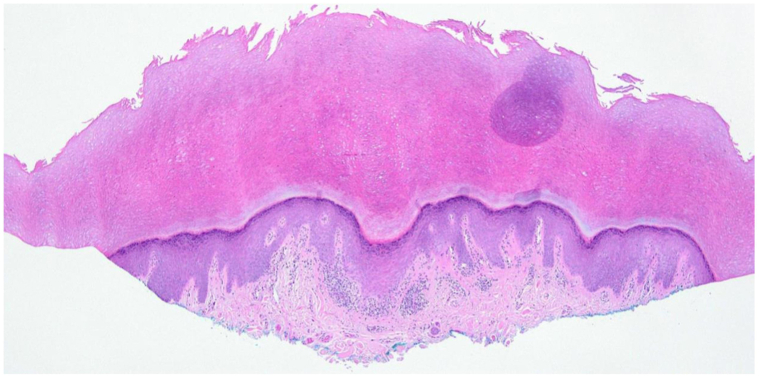
Skin biopsy from the right palm shows an epidermal invagination with compact orthohyperkeratosis extending beyond the stratum corneum (H&E, 40×). *H&E*, Hematoxylin and eosin.

**Fig 3:**
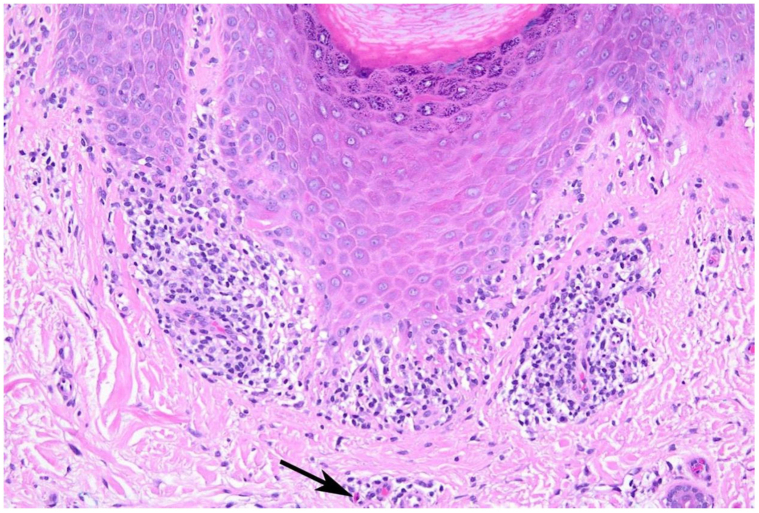
Higher magnification shows a lichenoid inflammatory infiltrate with dyskeratotic keratinocytes and superficial perivascular inflammation containing eosinophils (H&E, 200×). *Black arrow* highlights an eosinophil. *H&E*, Hematoxylin and eosin.

**Fig 4:**
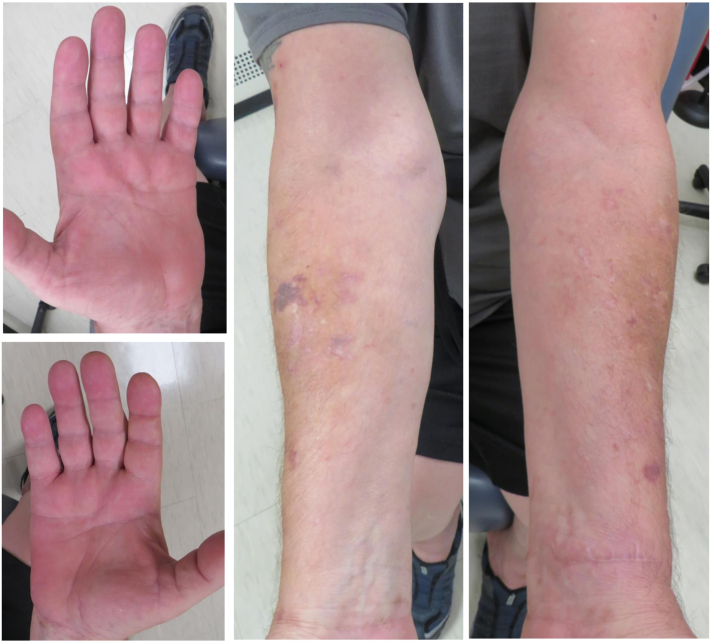
Images of bilateral palmar hands and ventral forearms taken at the patient’s July follow-up, demonstrating almost complete resolution of cutaneous eruptions following treatment with oral acitretin.

The patient reported painful lesions on his hands and feet that significantly impaired walking and day-to-day function. Initial treatments with clobetasol, triamcinolone 0.1%, and urea 40% creams were unsuccessful. In April, he was started on oral acitretin 25 mg daily. Follow-up in July revealed near-complete resolution of symptoms (Fig 4). His regimen was tapered to 25 mg every other day for 2 months, and he has since remained symptom-free.

## Discussion

Immune checkpoint inhibitors targeting PD-1 protein, such as pembrolizumab, are an effective and increasingly commonly used treatment for a variety of malignancies. As T-cell expression of PD-1 has been shown to promote neoplastic cell evasion of host immune systems, inhibitors of this receptor are able to restore cytotoxic T-cell function to improve survival in multiple malignancies, including melanoma, nonsmall cell lung carcinoma, and colorectal cancer.^7^^,^^9^

While these monoclonal antibodies tend to have an overall favorable safety profile, PD-1 inhibitors are associated with a distinct class of immune-related adverse events (irAEs) resulting from nonspecific immune activation.^5^^,^^10^ Cutaneous irAEs are among the most frequent, occurring in more than 30% of patients and typically manifesting as maculopapular eruptions, lichenoid reactions, eczema, or vitiligo.^5^^,^^10^ Dermatologic toxicities generally have an underlying immune etiology.^10^ irAEs generally occur within 1 month of starting treatment but may arise months later, occasionally even after treatment cessation.^7^^,^^9^ Recent evidence suggests that persistent toxicities, even after discontinuation of the immunotherapy, may be more common than previously believed as well.^7^^,^^9^

In our patient, the presence of both lichenoid dermatitis and punctate PPK raised consideration of a shared immunologic mechanism. While the lichenoid eruption is a recognized irAE, punctate PPK has not, to our knowledge, been previously associated with PD-1 blockade. Fradet et al hypothesized that PD-1 inhibitors may raise immunological response against UV exposure to result in transient hyperkeratosis.^5^ The co-occurrence of these 2 reaction patterns may reflect a broader spectrum of immune dysregulation induced by checkpoint inhibitors—one that includes aberrant keratinocyte turnover in addition to interface dermatitis.

Management of acquired punctate PPK focuses on identifying and addressing any underlying etiologies, including inflammatory conditions, infection, medications, and malignancy.^10^ Symptomatic care includes topical keratolytics such as salicylic acid, lactic acid, or urea; topical retinoids; debridement; psoralen plus UVA; and corticosteroids.^1^^,^^3^ For refractory cases, systemic retinoids like acitretin may be considered.^1^ Management in our case included acitretin for its anti-inflammatory and antiproliferative effects.

We recommend considering punctate PPK in the differential for cutaneous irAEs in patients undergoing treatment with pembrolizumab. We hope this report will assist oncology and dermatology specialists in timely diagnosis and treatment of PPK in patients who are receiving pembrolizumab immunotherapy. Further investigation may be warranted to elucidate potential immunologic or environmental triggers associated with punctate PPK in patients undergoing immunotherapy.

## Conflicts of interest

None disclosed.
